# Designing context-specific implementation strategies to improve water, sanitation, and hygiene in urban low-income settlements in Nigeria: outcome of stakeholders' collaborative approach

**DOI:** 10.3389/fpubh.2026.1742248

**Published:** 2026-03-05

**Authors:** Uchenna Ezenwaka, Iheomimichineke Ojiakor, Chinyere Okeke, Kenechukwu Ikeako, Joseph Nwankwo, Wilson Anyanebechi, Helen Elsey, Obinna Onwujekwe

**Affiliations:** 1 Health Policy Research Group, Department of Pharmacology and Therapeutics, University of Nigeria, Enugu, Nigeria; 2 Department of Health Administration and Management, Faculty of Health Sciences and Technology, University of Nigeria, Enugu, Nigeria; 3 Department of Parasitology and Entomology, Faculty of Biological Sciences, Nnamdi Azikiwe University, Awka, Nigeria; 4 Department of Health Sciences, Hull and York Medical School, University of York, York, United Kingdom

**Keywords:** collaborative approach, interventions, low-income settlements, multi-sectoral, Nigeria, stakeholder engagement, wash

## Abstract

**Background:**

Urban low-income settlements in Nigeria face significant challenges in accessing Water, Sanitation, and Hygiene (WASH) services, exacerbating health disparities and poverty. Collaborating to design effective and sustainable solutions is crucial for improving WASH outcomes. This study aims to present the process and outcomes of co-designing context-specific implementation strategies of WASH interventions in urban low-income settlements in Anambra State, Nigeria.

**Methods:**

The study employed a stakeholder-driven co-design process involving community members, researchers, and policymakers from both health and non-health sectors to develop context-specific implementation strategies for WASH interventions. Four workshops, fifteen in-depth interviews, and one FGD were conducted with these stakeholders. Outputs from the workshops and interviews were analyzed based on the Proctor implementation strategies framework.

**Results:**

Seventeen WASH interventions focusing on health education, advocacy, capacity building, and community-led activities were identified. Their key implementation strategies included the definition of each intervention, the actors that will be involved in the implementation, targets, actions, temporality, dose, and the implementation research outcome (IRO) measurements. Prioritization results informed the co-design of implementation strategies of top-prioritized WASH interventions. Stakeholders perceived the developed strategies as highly acceptable, feasible, and relevant to the community's needs. They believed that these would be adopted by end-users and have the potential to make an impact in their community when implemented due to the cultural appropriateness and potential to address specific community challenges. Enablers of the process included strong community participation, inclusion of diverse and experienced stakeholders, and use of local evidence in decision-making. Stakeholder management, including identifying and engaging appropriate individuals, coordinating schedules, and managing time demands, posed significant challenges. Key lessons learned indicate that leveraging informal networks, adapting local evidence approaches, and cultural sensitivity are crucial in designing interventions and navigating success.

**Conclusions:**

The study underscores the importance of a collaborative approach in designing effective and sustainable implementation strategies of WASH interventions for urban low-income settlements toward the achievement of SDGs 3 and 6. The findings underscore the need to integrate collaborative and context-specific approaches into WASH policy and programming.

## Background

Inadequate access to water, sanitation, and hygiene (WASH) affects about one-third of the global population, predominantly in low- and middle-income countries (LMICs), compromising health, dignity, and wellbeing (1). Millions of people in LMICs lack basic WASH services, with urban low-income settlements being disproportionately affected due to rapid urbanization, poor infrastructure, and limited access to essential services (2–4).

In Africa, approximately 70% of water schemes in urban low-income settlements are non-functional, resulting in poor access to clean and safe water (5). Due to poor WASH practices, about 28% of the population in this region still practice unsafe sanitation behaviors such as open defecation. Improved WASH practices have the potential to reduce the prevalence of diseases such as schistosomiasis, cholera, diarrhea, polio, and typhoid, which are prevalent in most sub-Saharan African countries (5). In Nigeria, the situation is severe, with over 60% of the population lacking basic sanitation facilities and more than 30% lacking access to improved water sources (6). Inadequate access to WASH services affects health, economic productivity, and environmental sustainability. For example, diarrhea is a leading cause of illness and death in Nigeria, with WASH-related factors playing a major role in this burden (7).

Ensuring universal access to WASH is a pressing public health concern. The Sustainable Development Goal 6 (SDG 6) aims to achieve universal access to clean water and sanitation by 2030. Despite this goal, significant inequalities persist, particularly in urban low-income settlements and rural communities in LMICs (5, 8).

Studies have shown that community-based interventions such as health education, behavior change communication, and capacity building of the community health workers to improve WASH significantly reduce the risk of most WASH-borne diseases, such as diarrheal, typhoid fever, dysentery, ARI, among others, that contribute to the high child mortality cases in informal urban settlements (9–13). It is, therefore, essential to develop effective and sustainable context-specific WASH interventions and strategies that take into account the unique social, cultural, and environmental factors influencing WASH practices in urban low-income settlements.

An effective approach to improving WASH services toward achieving SDG 6 is through collaborative approaches to designing intervention strategies adapted to the specific circumstances of each urban poor neighborhood with key stakeholders, including local governments, civil society, NGOs, and community members (6). Co-designing intervention implementation strategies is a collaborative and creative approach that brings together diverse stakeholders, including community members, researchers, and practitioners/implementers, to develop and implement public health interventions tailored to address specific community needs. In the context of urban low-income settlements, this approach can foster a sense of ownership, promote healthy behavioral change, and enhance sustainability and long-term impact by addressing unique challenges and access barriers (14–17). Collaborative engagement can facilitate the sharing of best practices, innovative solutions, and lessons learned, ultimately contributing to improved health outcomes and reduced inequalities among urban low-income settlement residents (14–17). Some key approaches to co-design include participatory research, collaborative design, and community-based participatory research, which share a commitment to involving stakeholders in the research process and taking their perspectives and needs into account (8). By working together, stakeholders can identify problems, design solutions, and test them in real-world settings, ensuring that interventions are context-specific and effective (8). Co-design involves working together to identify problems, design solutions, and test them in real-world settings (8). However, co-design can be challenging, particularly in complex urban settings with diverse stakeholders. Managing diverse perspectives and expectations, addressing power imbalances, and ensuring community engagement and participation can be significant hurdles (6, 8). Despite these challenges, co-design requires flexibility and adaptability, as interventions may need to be adjusted in response to changing contexts and circumstances (6, 8).

Effective design of an intervention relies on several key implementation strategies (18). To begin with, planning and collaboration are crucial, involving stakeholder engagement, building relationships, and fostering partnerships. Adaptation and tailoring are also essential in the co-designing implementation strategies of interventions, as they involve adapting the intervention to fit the specific context and needs of the stakeholders, with continuous feedback allowing for adjustments to be made as needed. Furthermore, support and resources are necessary to ensure the success of the intervention, including ongoing technical assistance, capacity building, and ensuring that necessary resources are available. Moreso, monitoring and evaluation are critical components of effective implementation. This includes process evaluation, which involves monitoring the implementation process to identify areas for improvement, and outcome evaluation, which assesses the effectiveness of the intervention. According to Proctor et al.'s framework (18), it's essential to consider several key elements when specifying implementation strategies. These include identifying the actors involved in the implementation process, the specific actions taken to implement the intervention, the context in which the implementation is taking place, the underlying mechanisms that drive the implementation, and the expected outcomes of the implementation (18).

Understanding how best to develop and deliver co-designed implementation strategies of interventions to achieve health improvements is necessary and has been explained in the implementation research (IR) (19). Implementation research (IR) is a specific scientific approach that evaluates the effectiveness of incorporating evidence-based interventions and policies into the health care system (20). IR focuses on contextual influences to implementing evidence-based interventions in health care systems and promotes the application, use, and sustainability of the interventions across various contexts (19, 20). Organizing policy-driven research, such as IR, supports the use of research findings to inform health practices to improve the effectiveness of service delivery and health care systems (20). IR looks at the audiences for the research, describes how the implementation occurs using the implementation outcome variables, and the strategies that support the delivery of the intervention/program (21). It also involves the integration of the findings and outcomes of the IR into policy and practice through the help of various stakeholders who should be identified and involved early in the intervention design stage. These stakeholders can affect various stages of the IR, such as influencing the intervention stages, including planning or designing implementation strategies, implementation, and monitoring stages of the intervention (22).

Stakeholders' collaboration in the co-designing of an intervention denotes different meanings for different individuals or groups. For the implementers, it helps in building their knowledge, skills, and capacity, and it produces evidence for policymaking (23). While for researchers, it helps in identifying IR outcomes and key findings that, although they are context-specific to the community, can be generalized to a wider population (24). Hence, the need for early initiation of the collaboration in a research setting, to give ample time for knowledge sharing between stakeholders, throughout the implementation of the research. Some studies have presented the need for collaboration in IR (16, 25). Others have shown the processes, enablers, and constraints to collaboration in research (26, 27), and a few have documented collaborations in developing implementation strategies within IR (22), but not much has been reported about collaborations to design an intervention to improve access to WASH, which is a major problem in the urban low-income settlements.

Collaboration is necessary in conducting IR, especially community-based ones, as such interventions are mostly influenced by the active engagement of a wide variety of local stakeholders, and their participation determines the success of the IR and prospects for its sustainability (16). It promotes acceptance and ownership of the intervention/program, builds trust among all parties involved, and improves acceptance of the research findings, especially if introduced early at the planning and strategy identification stages of the research (15, 16). Most collaborations involved partnerships among policymakers, officials from different ministries (such as Health, Environment, Information, and Education), community gatekeepers, NGOs, and academic researchers (22, 24). The contributions made by all these stakeholders to the design and implementation of an intervention will reflect their contextual issues, thereby ensuring that the emerging strategies are comprehensive, robust, and will meet the needs of the communities for which the intervention was designed (28) if the process is not interrupted by the researchers or funders. Choosing the wrong strategy or one that does not align with the local community context can impact the IR outcomes and undermine the success of the intervention (29).

A critical research gap exists in the design of WASH interventions in urban low-income settlements in Nigeria. Few studies have applied implementation science frameworks to develop context-specific interventions and implementation strategies in these settings, often neglecting complex contextual realities like informal governance, cultural norms, and power dynamics. This study addresses this gap by co-designing implementation strategies for WASH interventions in urban low-income settlements in Anambra State, Nigeria, using Proctor's framework through a stakeholder collaborative approach. The goal is to enhance access to WASH services and foster sustainable behavioral change. By generating evidence on context-specific strategies, this research aims to inform policy and practice, enabling stakeholders to design more effective and sustainable WASH programs that contribute to SDG 6 (Clean Water and Sanitation), SDG 3 (Good Health and Well-being), and SDG 11 (Sustainable Cities and Communities).

## Materials and methods

### Overview of the research project

The project aims to improve access to WASH in a major low-income settlement - Okpoko in Anambra State, Nigeria. Okpoko has been identified as a classic low-income settlement characterized by haphazard development, very high-density living, and a lack of public amenities and facilities/services, particularly WASH (30). The research employs a community-based participatory approach, guided by the Integrated Behavioral Model for WASH (IBM-WASH) (31, 32), the IR framework (18, 33), and the Theory of Change (ToC) framework (34). The project's objectives are multifaceted. Firstly, it seeks to assess the supply and demand-side factors that influence access to WASH among the urban poor population. Secondly, the project aims to determine the level of multi-sectoral involvement in WASH and identify ways to galvanize supportive actions from different sectors. This recognizes that improving WASH requires a collaborative effort from various stakeholders and sectors – policymakers/program implementers, community members, and researchers. Thirdly, based on the findings from the baseline assessment, the project aims to co-design and implement interventions designed to improve access to WASH facilities and promote improved health outcomes. These interventions will be tailored to address the specific needs and challenges identified in the baseline assessment. Lastly, the project will evaluate the effects of the interventions and identify factors that enable or constrain implementation. This will involve assessing the effectiveness and IR outcomes of the project in terms of acceptability, adoption, appropriateness, feasibility, fidelity, and sustainability. The project is expected to have several outcomes, including improved access to WASH facilities/services and improved health and wellbeing. By so doing, the project aims to contribute to achieving SDG 6 and improving the health and wellbeing (SDG 3) of the urban poor population in Anambra State, Nigeria.

### Study design/area

The study employs a stakeholder-driven approach, engaging community members, researchers, and policymakers/program implementers to design context-specific WASH solutions to achieve objective three of the research project. This study was conducted in Anambra State in the southern part of Nigeria, using the most populous low-income settlement, Okpoko, in Ogbaru LGA of the state. The low-income settlement has limited access to WASH services (35, 36) and a high prevalence of water-borne diseases (37).

### Study procedure and population

**Figure 1:**
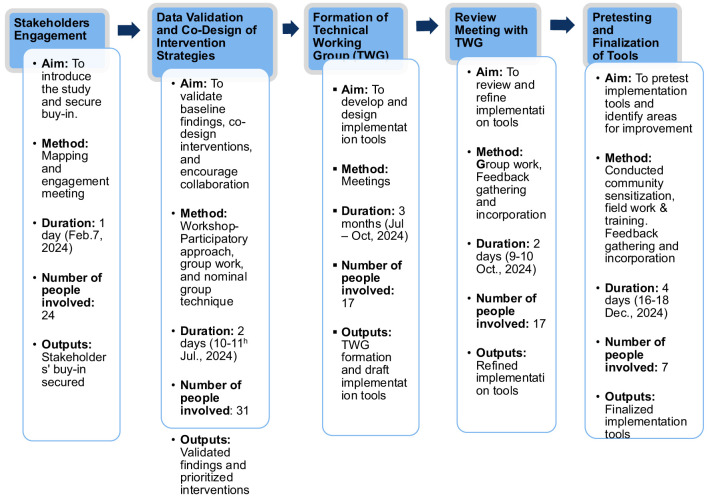
Schematic representation of the step-by-step process of co-designing the WASH intervention implementation strategies and tools.

The step-by-step process of co-designing the WASH intervention implementation strategies and tools is presented below and summarized in Figure 1.

#### Selection and engagement of stakeholders

The stakeholders were selected through an initial mapping exercise conducted by the researchers using a mapping template. Afterward, a stakeholders' engagement meeting was held on February 7, 2024, at the state capital, Awka, with 24 participants (9 males, 15 females) from various sectors/MDAs, including health, environment, education, information, public utility, and water resources, and community representatives. The meeting aimed to introduce the WASH project, secure buy-in, and gather information on WASH activities in the state. The meeting was facilitated by the research team.

#### Data validation and co-design of intervention strategies

Following a baseline assessment, a two-day workshop was held with stakeholders (policy makers, community members, and researchers) in the State. The workshop aims to: i) validate the findings from the baseline study; ii) co-design implementation strategies of WASH interventions, and iii) foster collaboration among stakeholders. The stakeholders were invited to participate in the workshop through formal letters delivered to their offices and communities. Thirty-one participants from diverse sectors attended the Awka workshop, validating findings and co-designing the WASH interventions. The workshop also provided a platform to harness the collective expertise and insights, and experiences of the stakeholders to develop effective solutions to the WASH challenges in the region.

The process began with a clear and concise presentation of baseline findings on the current state of WASH services in Ogbaru LGA (Okpoko), highlighting key challenges and barriers, and setting the stage for effective collaboration and targeted interventions. The workshop employed a highly participatory approach, where all stakeholders had an equal opportunity to contribute to discussions and decision-making. Power dynamics among the stakeholders were managed and encouraged through open discussion during co-design sessions, structured group work based on stakeholders' expertise/area of work, gender and local knowledge. This fostered an inclusive environment where all voices were heard. A consensus-based approach was used for decision-making, with divergent views resolved through discussion, prioritization, and iterative feedback loops.

Following the presentation and clarifications, participants were divided into three groups, each focusing on a specific thematic area: water, sanitation, and hygiene deliberated, suggested, and prioritized potentially feasible interventions based on the issues presented. Each group had 6–8 participants, and the discussions were moderated by appointed group leads. The research team was assigned to facilitate the discussions and ensure that the process was smooth and productive. Group composition was intentionally balanced based on knowledge, gender, and local knowledge, ensuring a mix of technical experts, community members, and policymakers. This structure encouraged collaboration and ensured diverse perspectives informed the WASH strategies. The water group, comprising community members, policymakers, and WASH experts; water officers, discussed water access challenges, equitable distribution, and sustainable management. The sanitation group, with community leaders, sanitation officers, and health experts, focused on infrastructure needs, behavioral change, and community-led initiatives. The hygiene group, consisting of teachers, community health workers, hygiene officers, and NGOs, explored hygiene promotion, education approaches, and cultural barriers. The Nominal Group Technique (NGT) was employed to facilitate this process, involving four structured stages. First, participants generated ideas silently, reflecting on WASH challenges and opportunities. Next, ideas were shared in a round-robin session, building on each other's suggestions. The ideas were then clarified and grouped for convergence. Finally, participants ranked their top preferences based on feasibility, impact, affordability, and sustainability, prioritizing interventions collectively.

During the plenary session, each group presented its proposed and ranked interventions. They received feedback from all participants, which informed a re-ranking process that culminated in the presentation of their final outputs. These final outputs (a set of prioritized interventions per WASH component) formed the basis for the development of implementation strategies and plans.

The final outputs were then used in a subsequent group work session to develop implementation strategies for each WASH component intervention, guided by the IR frameworks. These implementation strategies per component formed the second set of workshop outputs. Each group presented the outputs of their top three/four interventions in a plenary session, outlining detailed strategies and identifying implementation tools and potential facilitators to support the implementation of the interventions.

#### Formation of Technical Working Group (TWG)

At the end of the workshop, three TWGs were formed to assist the research team in developing and designing implementation tools for each intervention per component, as well as creating a monitoring and evaluation plan to track progress and assess effectiveness. The placement of the members of the TWG was based on area of expertise, position, and/or experience. The TWGs were composed of stakeholders from various sectors and operating at different levels. Each TWG had a specific focus: water, sanitation, or hygiene. The details of the TWG are shown in Table 1.

**Table 1:** The composition of the TWG for WASH interventions.

**Working group**	**Designation/position and organization**	**Category**	**Sector**	**Level of operation**	**Sex**
Water	Water Supply Officer, Rural Water Supply and Sanitation Agency (RUWASSA)	Policymaker/ Program implementer	Public Utility	State	Male
	Program Officer, Food Safety and Hygiene, State Ministry of Health (SMOH)	Program implementer	Health	State	Female
	President General, Okpoko Progressive Union	Community member	Community	Community	Male
	Researcher, University of Nigeria	Researcher	Academic Institution	Academic Institution	Female
	Researcher, University of Nigeria	Researcher	Academic Institution	Academic Institution	Male
Sanitation	Director of Environmental Health, Ministry of Environment (SMoE)	Policymaker	Environment	State	Female
	HOD Environmental Health and Sanitation	Policymaker	Environment	LGA	Female
	Health Facility Manager, Primary Health Center 1, Okpoko	Program implementer/Provider	Community	Community	Female
	Director of Public Health, SMoH	Policymaker	Health	State	Male
	Chief Security Officer, Okpoko Progressive Union	Community member	Community	Community	Male
	Researcher, University of Nigeria	Researcher	Academic Institution	Academic Institution	Female
	Researcher, University of Nigeria	Researcher	Academic Institution	Academic Institution	Male
Hygiene	Coordinator, WASH, SMOH, and Hygiene Officer, RUWASSA	Program implementer	Health and Public Utility	State	Female
	Coordinator, WASH, Ogbaru LGA	Program implementer	Health	LGA	Female
	Health Facility Manager, Primary Health Center 4, Okpoko	Program implementer/ Provider	Community	Community	Female
	Researcher, University of Nigeria	Researcher	Academic Institution	Academic Institution	Female
	Researcher, University of Nigeria	Researcher	Academic Institution	Academic Institution	Male

#### Review meeting with TWG

A two-day review meeting was held with the TWG to review and refine the developed WASH intervention implementation tools, gathering feedback on context and content, and incorporating suggestions for improvement to finalize the tools.

#### Pretesting and finalization of the tools

Afterward, a pretest of the implementation tools was conducted in Enugu low-income settlements over 4 days to identify areas for further improvement. The pretest helped to refine the tools and ensure they were effective in real-world settings. The feedback from the pretest was used to make necessary revisions to the tools. After the revision, all implementation tools were finalized and ready for implementation.

### Data collection and collation

Following the pretest and revision of the implementation tools, the data collection involved gathering/collating, and compiling the final outputs obtained from all workshops/processes. These include prioritized interventions, implementation strategies, a list of implementation tools, and a monitoring and evaluation plan. The collated outputs were then reviewed and validated by the TWGs and other stakeholders to ensure that they met the project objectives and were aligned with the needs of the target population.

A post-co-design process evaluation was done to reflect on and assess the effectiveness of the processes and lessons learned. In-depth interviews (IDIs) and focus group discussions (FGDs) were conducted with stakeholders and researchers. The IDIs were conducted with members of the TWGs, and FGD was done with the research team to elicit information on their experiences and perceptions of the engagement processes and outputs. A total of 15 stakeholders, including TWG members, were interviewed through IDI, while six research team members who facilitated the co-creation workshops were interviewed through FGD.

The interview/discussion guides explored the following: perceptions of the co-creation processes, strengths and weaknesses of the approach, suggestions for improvement, and feedback on the proposed interventions and implementation strategies, and tools. The IDIs were conducted by the four researchers who are experienced in qualitative data collection methods. Two researchers (a moderator and a notetaker) who are not part of the project but are experienced in qualitative interviews moderated the FGD session. The discussions were audio-recorded following respondents' permission and were transcribed verbatim. Each interview session lasted for about 40–45 min and was conducted in the respondents' offices.

### Data analysis and synthesis

The process outputs were organized into the themes of the IR implementation strategy framework (18). The framework's elements include the name of the intervention, its definition, the actors involved, the target population, the actions, the temporality, the dose, the IR outcomes (IRO), and the measurement. For the data analysis, we used thematic analysis with NVivo 12, employing multiple analysts to code data (using the codebook as shown in Table 2) and resolving discrepancies through consensus. The steps include: first, the transcripts underwent thorough editing and anonymization, where any identifying information was removed to safeguard participant confidentiality. Next, a thematic analysis approach was employed to code the transcripts, assigning relevant codes to emerging themes and patterns. These codes were then grouped into categories, allowing for the synthesis of findings and identification of key trends and insights (38). The analysis focused on identifying the strengths and weaknesses of the collaborative process, as well as lessons learnt for improvement. Finally, the findings were carefully interpreted within the context of the research objectives, informing the development of actionable recommendations to enhance the future co-creation process. Audit trails were maintained, and reflexivity exercises were conducted to ensure trustworthiness, allowing us to critically examine our own influence on the research process.

**Table 2:** CodeBook for qualitative analysis (IDI and FGD).

**Parent Node (Themes)**	**Child Nodes (Sub-themes)**	**Description of the code**	**Sample quotes**
Socio-demographic characteristics- FGD participants	• Stakeholders' level of operation • Position/Designation • Sex • Length of service in their current positions (years)	This code captures the level at which stakeholders involved in the process operate (state, LGA, community, researchers), their current posts held at work place or community that necessitated their selection and involvement/engagement in the process, their duration in their current role/post, which may reflect stakeholders' experience and expertise in WASH and the gender representation among stakeholders.	
Socio-demographic characteristics- IDI respondents	•Stakeholders' level of operation • Position/Designation • Sex • Length of service in their current positions (years)	This code captures the level at which stakeholders involved in the process operate (state, LGA, community, researchers), their current posts held at work place or community that necessitated their selection and involvement/engagement in the process, their duration in their current role/post, which may reflect stakeholders' experience and expertise in WASH and the gender representation among stakeholders.	
Stakeholders' perception	Perception	This code captures whether the stakeholders had a negative or positive view of the process and outcomes, which could indicate satisfaction or dissatisfaction with the approach and results.	
	Sense of Ownership	This code captures whether the community leaders/members felt invested in interventions due to involvement in development, leading to a sense of responsibility and commitment, as well as felt ownership due to an inclusive process.	
	Responsibility and Commitment	This captures whether the stakeholders took ownership and were committed to implementation, indicating a willingness to support the implementation of such interventions or not.	
Acceptability and adoption	High acceptability	This code captures whether the interventions were well-received by stakeholders due to their involvement in development, making them more likely to support and adopt the interventions.	
	Addressing Community Needs	This code captures whether the interventions were designed to address pressing issues and priorities identified by the community, increasing their relevance and potential impact	
Potential impact	Positive Impact	This code captures whether the interventions had the potential to improve community lives, addressing key challenges and improving WASH outcomes for residents.	
	Sustainability	This code captures using needs baseline assessment data contributed to lasting impact, as interventions were tailored to address specific, identified needs.	
Enablers	Inclusivity and Transparency	This code captures the involvement of stakeholders and being transparent throughout the process, which built trust and credibility among stakeholders, facilitating cooperation and support.	
	Diverse Stakeholder Experience	This code captures whether the stakeholders valued learning from each other's expertise and experiences, contributing to a positive experience and increased engagement.	
	Use of Needs Assessment	This code captures using data from the baseline assessment ensured interventions addressed actual, identified needs, increasing their relevance and effectiveness.	
	Strong Community Participation	This code captures whether the active community engagement ensured interventions fit the local context, addressing specific challenges and priorities.	
Challenges	Identifying Right Stakeholders	This code captures the stakeholders required to balance competing interests and ensure representation to ensure inclusivity and minimize conflicts.	
	Coordinating Stakeholder Schedules	This code captures whether managing schedules and the availability of stakeholders was challenging, requiring flexibility and persistence to ensure engagement	
	Managing Time Demands	This code captures whether engaging stakeholders took significant time and effort, requiring adaptability and flexibility to manage competing demands and priorities.	

### Ethics approval

The study was approved by the Ethics Committee of the University of Nigeria Teaching Hospital, Ituku Ozalla, Enugu (UNTH/HREC/2023/10/639) and the University of Leeds (MREC 23-013). Informed consent was obtained from all respondents before administering the questionnaire. This study was conducted following the Declaration of Helsinki.

## Results

### Socio-demographic characteristics of the respondents

The socio-demographic characteristics of respondents (Table 3) show diversity in levels, positions, and experience. The IDIs included state, community, and LGA stakeholders, while the FGD consisted of research institution participants. Females dominated the IDI group, while FGD had equal male-female representation. Respondents' positions varied, and most had 2–5 years of experience.

**Table 3:** Socio-demographic characteristics of the respondents.

**Variable (*****N*** = **15)**		**IDI *n* (%)**	**Variable**	**FGD *n* (%)**
Level of stakeholder	State	6 (40.0)	Research institution (University)	6 (100.0)
	LGA	3 (20.0)	—	—
	Community	6 (40.0)	—	—
Sex	Male	5 (33.3)	Male	3 (50.0)
	Female	10 (66.7)	Female	3 (50.0)
Position/designation	Director	2 (13.3)	Early researcher	4 (66.7)
	Coordinator/HOD	4 (26.7)	Mid-level	1 (16.7)
	Program managers/implementers	3 (20.0)	Senior researcher	1 (16.7)
	Community leaders	4 (26.7)	—	—
	Provider	2 (13.3)	—	—
Length of service in their current positions (years)	2–5	9 (60.0)	2–5	4 (66.7)
	6–10	6 (40.0)	6–10	1 (16.7)
	10 and above	0 (0)	10 and above	1 (16.7)

## Outcomes

### Prioritization of WASH Interventions

Table 4 presents proposed interventions and the prioritization outcomes of WASH interventions, ranked by importance and re-ranked by position. There were 17 proposed WASH interventions categorized into three components: water (seven interventions), Sanitation (six interventions), and Hygiene (four interventions). These interventions focus on advocacy, capacity building, health education, and community engagement to improve WASH practices. Capacity building and health education campaigns are cross-cutting interventions that appear across multiple WASH components. In the prioritization outcome, the ranking and re-ranking results reveal a consistent prioritization of advocacy, capacity building, and health education as top interventions, with high importance ratings, while also highlighting varying priorities across WASH components and consistency in low-priority interventions.

**Table 4:** Prioritization outcomes of WASH interventions by the stakeholders during the co-designing workshop.

**S/No**	**List of suggested interventions**	**Prioritization outcomes**	
		**Ranking by importance**	**Re-ranking by position**
**Water**			
1	Advocacy to government officials for more safer community water system and proper reticulation of existing boreholes	H	1st
2	Provision of household water treatment facilities	L	6th
3	Inspection of existing private boreholes and pure water companies, and instituting a penalty for owners of sub-standard boreholes	M	7th
4	Setting up a group/committee for monitoring, safeguarding, and maintaining public boreholes	M	4th
5	Health education campaign on safe water storage, treatment, and water-borne diseases and prevention in the communities	H	2nd
6	Capacity building of State, LGA, and community stakeholders on safe water supply chain management	H	3rd
7	Advocacy to government officials for a safer community water system and proper reticulation of existing boreholes	H	1st
**Sanitation**			
8	Construction of toilets/latrines in houses and institutions that do not have and mandatory evacuation of the sewage as at when due	L	6th
9	Advocacy visit to government offices and wealthy community members regarding the provision of refuse dumping sites and dumpsters, as well as the regular evacuation of waste in Okpoko.	H	4th
10	Institutionalization and enforcement of community sanitation rules, including toilet/latrine inspection and mandatory construction of toilets/latrine in all public institutions/places	H	3rd
11	Health education campaign on good sanitation practices and the effects of poor sanitation in the communities.	H	2nd
12	Advocacy for government engagement of more private waste evacuators	M	5th
13	Capacity building of State, LGA, and community stakeholders on good sanitation practices.	H	1st
**Hygiene**			
14	Enforcement of hand-washing stations in households and public institutions	L	4th
15	Health education campaign on proper hygiene knowledge, attitude, and practices in communities	H	3rd
16	Establishing WASH Clubs (as a hygiene promotion structure) to promote safe WASH in schools (WinS)	H	2nd
17	Capacity building of State, LGA, and Community stakeholders on good hygiene practices and promotion.	H	1st

The ranking keys (L = Low, M = Medium, H = High) indicate the priority level of suggested interventions, allowing for quick identification of areas of focus. Re-ranking in order of priority, with 1st being the highest priority, 2nd being medium priority, and 3rd being the lowest priority, and so on.

### Implementation strategies and tools

The outputs of the developmental process of the intervention implementation strategies for the three WASH components and their implementation tools are presented according to the IR strategies framework as described by Proctor et al. in Tables 5–7. The tables provide a detailed outline of the interventions, including actors involved, actions to be taken, and the evaluation metrics.

**Table 5:** Outputs of co-designed implementation strategies for water interventions developed by the stakeholders.

**Intervention name**	**Definition**	**Actors**	**Targets**	**Actions**	**Temporality**	**Dose**	**IRO & measurements**	**Key tools**
Advocacy to government actors for safer community water systems and proper reticulation of existing boreholes	This refers to advocating to government actors for safer community water systems and proper reticulation of existing boreholes involves strategic efforts to influence decision-makers and policies to prioritize and invest in safe water infrastructure toward improving access to safe and reliable water supply in the Okpoko community.	**State level** SMOH - Program Manager, WASH RUWASSA - Program Manager **LGA level** WASH Department **-**Coordinator, WASH **Community** President General Okpoko Progressive Union (OPU) and Woman leader, OPU **Civil Society Organization –** State representative **Media representative** **Researchers –** HPRG's Researchers	**State level** • Commissioner and Permanent Secretary, Ministry of Power and Water Resources (MoPWR), • Commissioner and Permanent Secretary, SMOH • Chairman, House committee on Power and water resources, and Speaker, Anambra state House of Assembly (who is from Ogbaru) **LGA Level** • Executive Chairman, Ogbaru LGA	• **Form an Advocacy Team/Technical Working Group (TWG)**, appointing a head, secretary, and designated presenters. Foster strong communication and networking among members to ensure effective collaboration. Propose meeting schedules, including dates, times, and venues. • **Conduct stakeholder mapping** to identify other key stakeholders relevant to water supply and management at state and LGA levels that may be included in the advocacy visit. • **Development of advocacy materials/messages-** advocacy briefs highlighting a clear and concise message, including a problem statement and how the problem affects Okpoko residents, and specific potential solutions to address the issue. • **Planning for the advocacy visits**, including meetings with the boundary partners: The mobilization of the actors; identification and provision of the necessary tools and materials needed for the advocacy visit; and developing a plan or schedule for the advocacy visits. • **Advocacy visits and follow-up plan-** Attend the advocacy meeting and deliver the meeting agenda as prepared; present advocacy materials and discuss the need for investment in water infrastructure. Facilitate discussions with the stakeholders to build consensus and support for the intervention, and follow up meetings with target groups. • **Reporting-** Document reports on the progress of advocacy activities and the outcomes of the advocacy and advocacy meetings • **Establish a monitoring plan -** Develop a framework to track the progress of government commitments of the intervention of a period of time.	**Early/initial phase**- Stakeholder mapping and analysis, development of advocacy brief, planning, and logistics for the advocacy visit. **Middle phase-** Advocacy visit and the implementation of a framework to track the progress of government commitments and ensure transparency. **Late phase -** Sustain Advocacy Efforts through follow-up meetings. Regularly document and report on the progress of advocacy activities and outcomes of meetings and workshops.	Each activity in the initial and middle phases will happen once. The report will also be done once. The sustainability of the advocacy efforts in the final phase will be done after 6 months.	**Acceptability** – The extent to which the advocacy intervention and its components are perceived as satisfactory and agreeable by the stakeholders. Number and quality of stakeholder engagement meetings, frequency and effectiveness of the advocacy visit, as well as the development of advocacy materials. **Adoption -** The extent to which the stakeholders implement or put in plan to implement the provision of more water systems in Okpoko. Policymakers buy-in to support safe community water systems and proper reticulation of boreholes. Secured government commitment to implementing the intervention (Report from the advocacy visits and IDIs interviews with the actors and target people).	Protocol, briefing notes and monitoring plan
Capacity building of State, LGA, and community stakeholders on safe water supply chain management	This intervention enhances the capabilities and skills of Community, LGA, and State-level stakeholders in the safe water supply management chain, including collection, storage, treatment, and prevention of waterborne diseases, through training and knowledge transfer.	**State level** **SMOH**- Program Manager WASH **RUWASSA-** Program Manager, RUWASSA **SPHCDA** – State Health Educator **Researchers –** HPRG's Researchers	**State Level** Program officers, RUWASSA; WASH Officers, SMOH; Water Officers, SOME; Program implementers, SPHCDA, and Social Mobilization Officer (SMO) **LGA Level** LGA WASH Officers; LGA Water Officers; LGA WASH Focal Person; LGA SMOs **Community Level** PG Okpoko, CSO, Woman leader, Health Facility Managers, Teachers from schools in Okpoko community	• **Develop/design intervention manuals and tools**, including manuals • **Pilot and finalize** the intervention/training manuals and tools • **Plan the capacity building training workshop:** Organize and plan logistics for training sessions, including venue selection and materials preparation and printing, developing the agenda, and task sharing. Also, secure approval from relevant organizations, and send invitation letters to trainees. • **Deliver** the capacity building workshop • **Evaluate** the training workshop	**Early/initial phase** -Planning and testing of intervention materials **Middle phase** -Full implementation of the training workshops. **Late phase** – Evaluating the intervention	Once - All actions in the initial phase will happen once, while the training will happen once for 3 days in the middle phase. Late phase will happen once during the end-line evaluation of the project.	• **Fidelity** - whether the training is implemented/delivered as designed in the original protocol • **Acceptability-** whether the training modules are agreeable or credible • **Appropriateness**- whether the intervention is perceived as fit or relevant in a particular setting or for a particular target audience or issue • **Coverage/reach** – whether the eligible/intended trainees are reached or have received the intervention (IDI and FGD)	Facilitators manual, participants manual, posters on water safety, Job aid on water safety, Pre and post-test, post-training evaluation form, agenda
Health education campaign on safe water storage, treatment, and water-borne diseases and prevention in the communities	This intervention provides community members with essential knowledge and skills related to safe water storage, treatment methods, and prevention of waterborne diseases, aiming to improve understanding, attitude, and practices related to water safety and reduce waterborne illnesses.	**LGA level** Trained LGA stakeholders: LGA WASH Officers; LGA Water Officers; LGA WASH Focal Person; LGA SMOs **Community level** Trained: community leaders PG Okpoko, CSO, Woman leader, WDC Chairmen, Health Facility Managers, Teachers from schools in Okpoko community **Researchers –** HPRG's Researchers	**Community level** Community members, School children/students, Teachers, Health facility staff	**Planning** – Development of health education materials and tools, including community sensitization flier and other IEC materials. **Mapping** of schools and identification of key community groups. **Community mobilization and sensitization-** engage community leaders/announcers through a town hall meeting to educate and raise awareness among the community members about the project. They can do this through town hall meetings, announcements in churches, health centers, etc. **Deliver the health education** in communities. **Evaluate** the campaign	**Early/initial phase –**planning of proposed activities, development of IEC materials, sensitization/mobilization activities. **Middle phase –**Delivery of health education sessions in schools and communities, continuous distribution of IEC materials **Late phase –** Evaluation of the activity to check the effectiveness of the education activities	Continuous community-wide engagement with frequent educational sessions and ongoing community interactions. **Coverage:** Extent of reach and engagement within the community. (IDI, FGD, Survey)	**Acceptability:** Level of satisfaction and approval from participants regarding the health education. **Adoption:** The extent to which the recommended water storage, treatment practices, and disease prevention measures are adhered to by the community. **Fidelity:** Degree to which the health education is delivered as planned. **Appropriateness:** Suitability of the educational content and methods for the target audience. **Adherence:** Consistency with which community members follow the recommended practices. Sensitization flyer, Posters on water safety, Job aid on water safety, Agenda, Attendant sheet	
Setting up a committee (BORESAFE) for monitoring, safeguarding, and maintaining public boreholes	This initiative establishes a community-led committee responsible for monitoring, safeguarding, and maintaining public boreholes, ensuring their functionality, safety, and sustainability through routine inspections, repairs, and community engagement, to improve access to clean water.	**Community level** Community leaders – PG, CSO, Youth Leader, Woman leader, among others **Researchers –** HPRG's Researchers	Public Borehole Safeguarding and Maintenance Committee (BORESAFE)	• **Develop SOP** for appointment/recruitment of members of the committee, itemizing committee composition, number of members, representation, and skills required. • **Develop TOR** for the detailing membership and their roles, purpose of the committee, roles and responsibility/task of the committee including identification of sources of funding and resources for maintenance and repairs, meeting schedule and procedures -set frequency, format and guidelines for meetings, MOU with Village local mechanisms (VILLOM), penalty for vandalizers and decision-making process, monitoring and reporting mechanisms. •** Convene a meeting** with community leaders to finalize the TOR • **Constitute and inaugurate** the committee • **Train** committee members on borehole monitoring and maintenance, and their roles. • **Create public awareness** about the committee and its task, and the penalty for offenders	**Early/initial phase –** development of SOP and TOR, planning meeting **Middle phase** – finalization of TOR and constitution and inauguration of BORESAFE committee **Late phase** – Evaluation of the activity	Once the committee is constituted and inaugurated	**Acceptability:** Level of satisfaction and approval from community members and the committee on SOP and TOR. **Adoption:** The extent to which the recommended tasks are followed and implemented. **Fidelity:** Degree to which the SOP and TOR are adopted and implemented. (monitoring report, IDI and FGD)	SOP, TOR, monitoring checklist, activity log book

Implementation strategies prioritized (based on feasibility and impact) water interventions based on feasibility and impact.

**Table 6:** Outputs of co-designed implementation strategies for sanitation interventions developed by the stakeholders.

**Intervention name**	**Definition**	**Actors**	**Targets**	**Actions**	**Temporality**	**Dose**	**IRO & measurement**	**Key tools**
Capacity building of State, LGA, and community stakeholders on good sanitation practices.	This intervention empowers state, LGA, and community stakeholders on principles and standards of proper sanitation through training, aiming to increase knowledge and improve implementation of sanitation programs.	**State level** **SMOH-** Director of Public Health, Program Manager, WASH **SOME** – Director, Environmental Health Services **SPHCDA** – State Health Educator **LGA Level** – HOD, Environmental Health **Researchers –** HPRG's Researchers	**State Level** Program officers, RUWASSA; Sanitation Officers, SMOH; Sanitation Officers, SOME; Program implementers, SPHCDA, and Social Mobilization Officer (SMO) **LGA Level** LGA WASH Officers; LGA Sanitation Officers; LGA WASH Focal Person; LGA SMOs **Community Level** PG Okpoko, CSO, Woman leader, Health Facility Managers, Teachers from schools in Okpoko community	• **Develop/design intervention manuals and tools**, including manuals • **Pilot and finalize** the intervention/training manuals and tools • **Plan the capacity building training workshop:** Organize and plan logistics for training sessions, including venue selection and materials preparation and printing, developing the agenda, and task sharing. Also, secure approval from relevant organizations, and send invitation letters to trainees. • **Deliver** the capacity building workshop • **Evaluate** the training workshop	**Early/initial phase** -Planning and testing of intervention materials **Middle phase** -Full implementation of the training workshops. **Late phase** – Evaluating the intervention	Once - All actions in the initial phase will happen once, while the training will happen once for 3 days in the middle phase. Late phase will happen once during the end-line evaluation of the project.	• **Fidelity** - whether the training is implemented/delivered as designed in the original protocol • **Acceptability-** whether the training modules are agreeable or credible • **Appropriateness**- whether the intervention is perceived as fit or relevant in a particular setting or for a particular target audience or issue • **Coverage/reach** – whether the eligible/intended trainees are reached or have received the intervention (IDI and FGD)	Facilitators manual, participants manual, posters on proper sanitation practices, Job aid on proper sanitation practices, Pre and post-test, post-training evaluation form, agenda
Institutionalization and enforcement of community sanitation rules, including toilet/latrine inspection and mandatory construction of toilets/latrine in all public institutions/places	This intervention establishes, inspects, and enforces sanitation rules and laws in communities and institutions, ensuring safe and accessible toilets/latrines, promoting a healthy environment, and reducing disease risk.	**State level** **RUWASSA** – Program Manager and Sanitation Officer **SMOE** – Director Environmental Health Services **LGA level** -Sanitation Officer, HOD, Environmental Health **Community leadership –** President General, CSO, Woman leader, Security agents (community-based vigilante group), existing community sanitation Task Force, Head of Landlord Association, ANSEPA **Researchers –** HPRG's Researchers	Landlords, Community members, churches, schools, markets, etc	• **Develop/review TOR for the existing sanitation task force committee** detailing membership and their roles, purpose of the committee, roles and responsibility/task of the committee including identification of sources of funding and resources for maintenance and repairs, meeting schedule and procedures -set frequency, format and guidelines for meetings, checklist for conduct sanitation assessment including toilets and bathroom in buildings, schools, churches inspection and identify institution without toilets/latrines, penalty for offenders and decision-making process, monitoring and reporting mechanisms. • **Convene a meeting** with community leaders and the sanitation task force to finalize and adopt the revised TOR • **Create public awareness** about the committee and its task, and the new penalty for offenders	**Early/initial phase –** development/review of TOR, planning meeting **Middle phase** – finalization of TOR **Late phase** – Evaluation of the activity	All actions in the initial and middle phase will happen once, while monitoring of sanitation violations will be every last Saturday of the month	**Acceptability** – To check the extent to which the stakeholders have accepted institutionalization and monitor sanitation practices in the communities. **Adoption:** to check the level of monitoring, supervision, adherence to sanitation laws, and penalization of defaulters happening in the community. **Fidelity** - how they adhere to the use of the materials provided for the monitoring and supervision activities. (Reports, IDI and FGD)	TOR, Register/report book, checklist, personal identification materials, e.g., face caps, apron, etc., personal protective equipment (PPE), e.g., apron, etc.
Health education campaign on good sanitation practices and the effects of poor sanitation practices in communities.	This intervention educates people about healthy sanitation behaviors, disease prevention, and the importance of good sanitation practices, aiming to increase knowledge and promote positive health outcomes.	**LGA level** Trained LGA stakeholders: LGA WASH Officers; LGA Sanitation Officers; LGA WASH Focal Person; LGA SMOs, HOD Environmental Health, HOD Health **Community level** Trained: community leaders PG Okpoko, CSO, Woman leader, WDC Chairmen, Health Facility Managers, Teachers from schools in Okpoko community **Researchers –** HPRG's Researchers	**Community level** Community members, School children/students, Teachers, Health facility staff	• **Planning** – Development of health education materials and tools, including community sensitization flyer and other IEC materials. • **Mapping** of schools and identification of key community groups. • **Community mobilization and sensitization-** engage community leaders/announcers through a town hall meeting to educate and raise awareness among the community members about the project. They can do this through town hall meetings, announcements in churches, health centers, etc. • **Deliver the health education** in communities. • **Evaluate** the campaign	**Early/initial phase –**planning of proposed activities, development of IEC materials, sensitization/mobilization activities. **Middle phase –**Delivery of health education sessions in schools and communities, continuous distribution of IEC materials **Late phase –** Evaluation of the activity to check the effectiveness of the education activities	Continuous community-wide engagement with frequent educational sessions and ongoing community interactions.	**Acceptability:** Level of satisfaction and approval from participants regarding the health education. **Adoption:** The extent to which the recommended water storage, treatment practices, and disease prevention measures are adhered to by the community. **Fidelity:** Degree to which the health education is delivered as planned. **Appropriateness:** Suitability of the educational content and methods for the target audience. **Adherence:** Consistency with which community members follow the recommended practices. **Coverage:** Extent of reach and engagement within the community. (IDI, FGD, Survey)	Sensitization flyer, Posters on proper sanitation practices, Pamphlet on good sanitation practices, Job aid on proper sanitation practices, Agenda, Attendant sheet
Advocacy visit to government offices and wealthy community members on the provision of refuse dumping sites and dumpsters, and regular evacuation of waste in Okpoko.	This intervention advocates for effective waste management services, including the provision of refuse dumping sites, dumpsters, and regular waste evacuation in Okpoko, to promote proper waste disposal practices and reduce environmental and health risks.	Community leadership, LGA Environmental health officers, sanitation task force, head of landlord association, ANSEPA, ASWAMA HPRG researchers	**State and LGA** policymakers, Landlords association, social clubs, and philanthropists etc in Okpoko wards	• **Develop a protocol that will** provide a detailed guide on the processes, procedures, and standards for implementing the advocacy intervention, ensuring consistency and quality in advocacy activities • **Collaborate with community leaders** to advocate to the government officials to secure land and resources for waste management infrastructure eg, refuse dumping site, and install receptacles/dumpsters in designated locations in Okpoko. • **Advocate for** a regular waste evacuation schedule	**Early/initial phase** – To provide a detailed guide on the processes, procedures, and standards for implementing the advocacy intervention, ensuring consistency and quality in advocacy activities **Middle phase** – Collaborate with community leaders to pay advocacy visits to government officials to secure land and resources for waste management infrastructure and a regular waste evacuation schedule. **Late phase** – Encourage follow-up advocacy	All actions will happen once, but a follow-up might be required.	**Acceptability** – To check the extent to which the stakeholders have accepted to conduct advocacy visits to relevant bodies for a designated dump site and acquire the required infrastructure. **Adoption:** to check if the site was located, bins were provided, and refuse is being cleared as at when due. **Fidelity** - how they adhere to the clearing of the refuse as scheduled. (Report, IDI, Checklist)	Protocol, Advocacy briefing notes

Implementation strategies prioritized (based on feasibility and impact), sanitation interventions based on feasibility and impact.

**Table 7:** Outputs of co-designed implementation strategies for hygiene interventions developed by the stakeholders.

**Intervention name**	**Definition**	**Actors**	**Target**	**Actions**	**Temporality**	**Dose**	**IRO & measurement**	**Key tools**
Capacity building of State, LGA, and Community stakeholders on good hygiene practices and promotion.	This intervention develops and strengthens the capacity and skills of WASH stakeholders at the state, LGA, and community levels on good hygiene practices and principles through training, enabling them to promote and advocate for good hygiene practices.	**State level** **SMOH**- Program Manager WASH and Food and Hygiene Safety Officer **RUWASSA-** Program Manager **SPHCDA** – State Health Educator **Researchers –** HPRG's Researchers	**State Level** Program officers, RUWASSA; WASH Officers, SMOH; Hygiene Officers, SOME; Program implementers, SPHCDA, and Social Mobilization Officer (SMO) **LGA Level** LGA WASH Officers; LGA Hygiene Officers; LGA WASH Focal Person; LGA SMOs **Community Level** PG Okpoko, CSO, Woman leader, Health Facility Managers, Teachers from schools in the Okpoko community	• **Develop/design intervention manuals and tools**, including manuals • **Pilot and finalize** the intervention/training manuals and tools • **Plan the capacity building training workshop:** Organize and plan logistics for training sessions, including venue selection and materials preparation and printing, developing the agenda, and task sharing. Also, secure approval from relevant organizations, and send invitation letters to trainees. • **Deliver** the capacity building workshop • **Evaluate** the training workshop	**Early/initial phase** -Planning and testing of intervention materials **Middle phase** -Full implementation of the training workshops. **Late phase** – Evaluating the intervention	Once - All actions in the initial phase will happen once, while the training will happen once for 3 days in the middle phase. The late phase will happen once during the end-line evaluation of the project.	**Fidelity** - whether the training is implemented/delivered as designed in the original protocol **Acceptability-** whether the training modules are agreeable or credible **Appropriateness**- whether the intervention is perceived as fit or relevant in a particular setting or for a particular target audience or issue **Coverage/reach** – whether the eligible/intended trainees are reached or have received the intervention (IDI and FGD)	Facilitators manual, participants manual, posters on proper hygiene practices, Job aid on proper hygiene practices, Pre and post-test, Post-training evaluation form, agenda
Health education campaign on proper hygiene knowledge, attitude, and practices in communities	This intervention educates community members and institutions on proper hygiene practices, the importance of good hygiene, and WASH-borne diseases, aiming to improve knowledge, attitude, and behavior toward hygiene and prevent diseases	**LGA level** Trained LGA stakeholders: LGA WASH Officers; LGA Hygiene Officers; LGA WASH Focal Person; LGA SMOs, HOD Environmental Health, HOD Health **Community level** Trained: community leaders PG Okpoko, CSO, Woman leader, WDC Chairmen, Health Facility Managers, Teachers from schools in Okpoko community **Researchers –** HPRG's Researchers	**Community level** Community members, School children/students, Teachers, Health facility staff	• **Planning** – Development of health education materials and tools, including community sensitization flyer and other IEC materials. • **Mapping** of schools and identification of key community groups. • **Community mobilization and sensitization-** engage community leaders/announcers through a town hall meeting to educate and raise awareness among the community members about the project. They can do this through town hall meetings, announcements in churches, health centers, etc. • **Deliver the health education** in communities. • **Evaluate** the campaign	**Early/initial phase –**planning of proposed activities, development of IEC materials, sensitization/mobilization activities. **Middle phase –**Delivery of health education sessions in schools and communities, continuous distribution of IEC materials **Late phase –** Evaluation of the activity to check the effectiveness of the education activities	Continuous community-wide engagement with frequent educational sessions and ongoing community interactions.	**Acceptability:** Level of satisfaction and approval from participants regarding the health education. **Adoption:** The extent to which the recommended water storage, treatment practices, and disease prevention measures are adhered to by the community. **Fidelity:** Degree to which the health education is delivered as planned. **Appropriateness:** Suitability of the educational content and methods for the target audience. **Adherence:** Consistency with which community members follow the recommended practices. **Coverage:** Extent of reach and engagement within the community. (IDI, FGD, Survey)	Sensitization flyer, Posters on proper hygiene practices, Pamphlet on good hygiene practices, Job aid on proper hygiene practices, Agenda, Attendant sheet
Establishing WASH Clubs (as a hygiene promotion structure) to promote safe WASH in schools (WinS)	This intervention establishes school-based hygiene promotion structures (WASH Club), trains school hygiene peer educators and teachers to promote safe WASH practices, foster school ownership, and encourage collective actions and behavior change toward improved hygiene practices among students.	**LGA** - Trained stakeholder- WASH Coordinator, LGA WASH health focal person, LGA Health Educator, LGA Hygiene Officer, **Schools-** Trained Teachers, and school Principal of schools, ASUBEB – School Health Program Manager **Researchers –** HPRG's Researchers	Secondary school students, Teachers	• **Develop protocol** for establishing school clubs, including goal and objectives, scope, members and responsibilities, steps and club structure, meetings and activities, etc. • **Club Formation-** Establish the WASH club in the school and define its purpose, objectives, and roles. • **Membership Recruitment-** Recruit student members and identify leaders or peer educators. • **Inauguration-** Officially inaugurate the WASH club, marking its launch and introducing it to the school community. Conduct teachings on safe WASH practices. • **Monitoring and Evaluation-** Establish a system to monitor and evaluate the club's activities and impact. • **Sustainability Planning -** Develop strategies to sustain the club's activities and ensure long-term impact.	**Early/initial phase –** development of protocol, planning meeting **Middle phase** – formation of the club and inauguration of the School WASH Club. Training on safe WASH practices **Late phase** – Evaluation of the activity	Actions in the middle and late phases should be conducted quarterly	**Acceptability:** Level of satisfaction and approval from schools, peer educators, and teachers **Adoption:** The extent to which the recommended protocol and responsibilities are followed and implemented. **Fidelity:** Degree to which the protocol is adopted and implemented. (Report, IDI and FGD)	Protocol, monitoring checklist, activity log book, pamphlets on safe WASH, Job aids on safe WASH, posters on WASH, club register

Implementation strategies prioritized (based on feasibility and impact) hygiene interventions based on feasibility and impact.

### Stakeholders' perception of the process and the outcomes

#### Acceptability and adoption of the interventions and their implementation strategies

The stakeholders perceived the process and the outcomes positively. They found the proposed interventions implementation strategies highly acceptable, largely due to the inclusive and transparent process used to develop them. Community members felt a sense of ownership over the proposed interventions, having been involved in every stage of the exercise. This fostered a sense of responsibility and commitment among community members. The stakeholders believed that the interventions addressed specific community needs and priorities, making them more adoptable.

“*All the interventions are good and will change the lives of the community members*.”(IDI, LGA-Level Stakeholder)

The stakeholders' positive perception of the intervention and its implementation strategies is likely to enhance its adoption and sustainability. They felt that by involving the community in the development and implementation of the intervention, stakeholders had fostered a sense of ownership and responsibility among community members.

“*Yes, the participation of the community members, the teachings, and demonstrations were more than enough. The information regarding good WASH practices was acquired from the teachings, and surely, the community members will do well to apply them.”* (IDI, LGA-Level Stakeholder)

#### Potential to have impacts

Stakeholders stated that the intervention demonstrates the potential to positively impact the lives of low-income settlement residents when implemented, citing the following reasons: i) Incorporating findings from baseline assessment improves the chances of positive outcomes and sustainability.

“*Let me tell you, developing these interventions based on the Okpoko WASH issues is very good and will help them.” “It means that you heard from the horse's mouth…. You are solving their problems directly*.” (IDI, state-level stakeholder)

### Contextual influences of the collaborative process

Enablers

Several factors enabled the success of the WASH interventions development processes. These include:

#### Inclusivity and transparency of the process

The stakeholder stated that the process was not only inclusive and transparent but also intensive, fostering trust and credibility. Respondents mentioned that all the key stakeholders, including the community leaders/members, were represented and their voices heard in the workshops. The community's active participation throughout the process was stated to have increased the likelihood of adoption and sustainability of the interventions when implemented. The community members reported that through the engagement, they have been encouraged to take ownership of their WASH challenges and work collaboratively to implement the solutions/interventions.

“*The stakeholders that participated in the workshops were rightfully selected.”* (IDI, LGA level stakeholder)

“*I also want to chip in on the co-creation brought in the various groups. We had people from all levels. We had the states, the local government, and the community people represented in each of these three major areas in WASH…..There is inclusiveness and representation. Different levels of the stakeholders were at that workshop, creating/developing, or suggesting interventions and designing the implementation strategies.”* (FGD, Researchers)

#### Diverse and Positive Stakeholder Experience

Stakeholders reported having had a positive experience, characterized by cross-learning, increased knowledge, a sense of ownership, and satisfaction, which contributed to their engagement and commitment. Stakeholders appreciated the opportunity to meet and learn from each other's expertise and experiences during the process.

“*So in the sanitation where I was, I was observing them. We could see that there was no senior to this. When the local government people were more in charge of most of the activities when they shared their experiences. We hear the States people say, Is it how you people do it? So you could see that, cross learning was happening even at that level.”* (FGD, Researchers)

#### Use of needs assessment findings

The respondents felt that using the findings from the baseline assessment to develop interventions and their implementation strategies was quite useful and relevant. According to them, it ensured that the intervention strategies developed were tailored to specific needs and context. This approach helped identify the most pressing WASH challenges faced by the community and informed the successful development of targeted interventions.

“*It is a true and welcomed development because it goes a long way in enabling the community to identify their health challenges concerning* WASH activities”, “*The baseline findings is the actual reflection of the needs of Okpoko community, mostly that of sanitation” “The findings from the baseline assessment truly represents the needs of Okpoko people especially that of water and refuse bins.”* (IDI, LGA-Level Stakeholder)

#### Strong community participation

Strong community participation emerged as a key enabler of the process, facilitating effective collaboration, fostering trust, and ensuring that interventions and their implementation strategies were tailored to meet the specific needs and contexts of the community. Through active engagement and involvement, community members provided valuable insights, contributed to decision-making, and helped shape the development of implementation strategies of the WASH interventions that were responsive to local priorities and realities. One of the community members said,

“*So, in the past, we identified the need for a water source, then UNICEF built boreholes here for the community, the government also provides, but the thing is that they hand it over to the community stakeholders to manage and protect it. So, now we will identify our needs and hope that you people will provide them*.” (FGD, Males, Community Stakeholders)

Then another community member said,

“*What we need is plenty, we need more training so that we can pay attention to our environment and ensure good hygiene and clean drinking water is available in our community*.” (FGD, Females, Ward four Community Stakeholders)

Challenges

The development of the WASH interventions faced several challenges during the collaborative process that impacted the process. These challenges were:

#### Identifying the right stakeholders

Identifying and selecting the right stakeholders was a significant challenge. This required careful consideration to ensure inclusivity and manage conflicts of interest from the stakeholders. This challenge affected the process by requiring additional time and effort to identify and engage the right stakeholders,

“*I think I would say the first challenge was being able to identify all stakeholders that are supposed to be involved… making sure that no group was left behind… Because any group you miss has an issue… they will not be willing to cooperate fully.”* (FGD, Researchers)

However, the careful consideration given to stakeholder selection ultimately contributed to the development of a more inclusive and effective intervention.

#### Coordinating with stakeholders' schedules

The process of coordinating with busy stakeholders was a bit tiring because it required persistence, patience, and adaptability. The research team had to follow up with calls and appointments to ensure that stakeholders remained engaged and informed throughout the process.

“*You still need to call, book appointments, make sure you follow up, wait for some of them who might be very busy, like the PG. You have to go many times… Though they come to your activities, it does not guarantee that they will always answer you when you call on them. So you need to be persistent, you need to be patient, you also need to bring yourself down to their level.”* (FGD, Researchers)

This impacted the process by requiring flexibility and creativity in scheduling and communication.

#### Managing the time demands of stakeholder engagement

The time-demanding nature of stakeholder engagement was a significant challenge, consuming considerable time and resources. This included the time required to engage stakeholders, build relationships, and facilitate communication.

“*So working with stakeholders with different interests and backgrounds is quite challenging… getting them to come and sit down and then discuss issues around WASH is quite challenging… You need to have time and consider their time constraint in all the activities you do because you need to carry these stakeholders along.”* (FGD, Researchers)

### Lessons learnt from the engagement

The lessons learned from this co-design process reflect broader patterns observed across co-design practice, highlighting key factors that contribute to successful intervention development. First, engaging stakeholders from diverse sectors and levels was instrumental in tailoring interventions to specific community needs and context, fostering synergy and cross-learning among stakeholders, as seen in similar co-design contexts. This collaborative approach enhances stakeholders' knowledge and skills, promoting effective WASH program development. In addition, understanding and respecting community norms, values, and practices proved crucial for acceptability and adoptability, building trust and rapport with community members. More so, managing diverse stakeholders with different interests and backgrounds poses challenges, requiring careful coordination, detailed mapping, follow-up, and conflict management to ensure interventions are inclusive, equitable, and sustainable. Notably, community involvement and ownership emerged as critical for sustainability and success, empowering community members to devise solutions tailored to their needs, increasing the likelihood of adoption and impact. Finally, capacity-building interventions are necessary to address knowledge gaps, particularly among new stakeholders or those lacking experience in WASH program planning and implementation, enabling them to contribute effectively. These insights from the process underscore the importance of nuanced, context-sensitive co-design approaches prioritizing stakeholder engagement, community involvement, and capacity-building, aligning with lessons from other co-design contexts.

## Discussion

This study demonstrates an inclusive and participatory process in co-designing context-specific implementation strategies for interventions to improve access to WASH services in urban low-income settlements in Nigeria. The prioritization of WASH interventions focusing on health education, advocacy, capacity building, and community-led activities for sanitation services and protection, safeguarding, and maintenance of public boreholes underscores the importance of a multi-faceted approach in addressing WASH challenges. These findings are consistent with existing literature that highlights the need for comprehensive WASH interventions that address the complex and interconnected nature of water, sanitation, and hygiene (39).

The prioritization of community-led activities for sanitation services and protection, in particular, highlights the critical role that communities can play in ensuring the sustainability and effectiveness of WASH interventions (40). Evidence from rural Ghana trials over two decades similarly shows that interventions designed and enforced by community bodies maintain higher functionality over time compared to externally managed programs (16). For Nigerian urban low-income settlements, institutionalizing such committees within local associations and linking them to modest budgetary allocations could ensure long-term sustainability and accountability. The implications of these findings for policy and practice are significant. Policymakers and practitioners should prioritize community-led approaches and ensure that interventions are tailored to specific community needs. This may involve investing in capacity building and training for community members and leaders, as well as ensuring that interventions are culturally sensitive and responsive to local contexts (40). By prioritizing community-led approaches and tailoring interventions to specific community needs, policymakers and practitioners can make progress toward achieving SDG 6. Interventions, when implemented can play a critical role in reducing the risk of water-borne diseases and promoting overall health and wellbeing (SDG 3) (41).

Stakeholders' perceptions of the developed interventions as highly acceptable, feasible, and relevant to the community's needs are encouraging and suggest that involving stakeholders in the design process enhances the potential for successful implementation and impact. Some strategies, such as community-led hygiene education and stakeholder engagement, are immediately actionable within existing resource constraints. These strategies leverage local capacity and can be integrated into ongoing WASH programs and activities. However, others, like infrastructure upgrades and financing mechanisms, require policy changes and substantial investment. Hence. we anticipate potential barriers related to governance structures, financing, and long-term sustainability, which will be addressed through stakeholder engagement, phased implementation, and alignment with national and state-level WASH priorities. Our findings are consistent with existing literature that highlights the importance of stakeholder engagement and participation in WASH intervention design and implementation (42).

The perceived cultural appropriateness and potential to address specific community challenges of the developed interventions are particularly noteworthy, as they suggest that the interventions are well-suited to the local context and are likely to be effective in addressing community needs. The finding implies an increased likelihood of successful implementation and impact, and makes progress toward achieving SDG 6 and SDG 3 if well implemented. A study in rural Tanzania demonstrated that contextualized hygiene promotion achieved better uptake than standardized packages, reinforcing that tailoring to baseline findings, as in this study, is not only culturally sensitive but essential for feasibility. Policymakers should therefore mandate baseline participatory assessments as a precursor to designing urban low-income settlement interventions, making “evidence of local fit” a funding criterion (14).

The co-design approach's key enablers- community participation, diverse stakeholders' inclusivity, and local evidence use underscore the importance of collaboration and evidence-based strategies in WASH intervention design and implementation. By harnessing diverse perspectives and expertise, this approach yielded tailored solutions that fostered stakeholders' ownership and enhanced intervention relevance and feasibility, aligning with existing literature on co-design's value in public health (22, 42, 43). More so, using local evidence in decision-making ensured interventions were tailored to community needs and contexts, hence the importance of investing in mobilization and ensuring data informs interventions. Data-driven approaches have revolutionized public health, facilitated targeted interventions and improved outcomes, as seen in examples like US opioid epidemic management (20% reduced mortality), Rwanda's maternal health program (30% declined mortality), and India's TB program (15% reduced prevalence) (44–49).

However, in this study, the complexities of engaging stakeholders in the program design posed challenges—identifying the right people, coordinating their schedules, and managing time demands, emphasizing the need for careful planning and resource investment (14–16). Similar barriers have been reported by the Nigeria Implementation Science Alliance (NISA), where aligning multi-sectoral actors required iterative negotiation and flexible facilitation (26). This emphasizes the need for institutionalizing multi-sector WASH platforms while also funding them as part of a routine planning strategy.

One limitation is that the findings may be specific to the particular low-income settlement context in Anambra state, Nigeria, and may not be generalizable to other low-income settlements or settings. Additionally, the stakeholders' responses may have been influenced by social desirability bias, where they provided answers that they thought were expected of them rather than their true opinions. However, the findings from the baseline assessment in the low-income settlements validated their responses. Furthermore, the focus on a single low-income settlement setting may limit the external validity of the findings.

Despite these limitations, the study has several strengths. Engagement with diverse stakeholders from various levels and sectors, including community leaders, added depth and richness to the findings, ensuring that a wide range of perspectives was represented. Also, the use of baseline assessment findings to inform intervention design ensured that the interventions were grounded in local evidence and tailored to the specific needs of the community. Moreover, the comprehensive evaluation of stakeholder perceptions of the whole process and outcomes provided a thorough understanding of the strengths and weaknesses of the intervention design process. Lastly, the presence and involvement of low-income settlement community leaders in the workshops and interviews ensured that the study was community-driven and that the findings were relevant and applicable to the local context.

## Conclusion

This study demonstrates the effectiveness of a stakeholder-driven co-design process in developing context-specific interventions and implementation strategies to improve access to WASH in Anambra State, Nigeria. This study highlights key lessons for designing WASH interventions in urban low-income settlements, including the importance of inclusive participation, community-led approaches, cultural sensitivity, and iterative planning. The findings provide valuable insights for policymakers, researchers, and practitioners seeking to develop effective and sustainable WASH solutions in similar settings. Ultimately, this co-design approach has the potential to inform scalable and impactful WASH interventions, contributing to improved health outcomes and reduced disparities in urban low-income settlements.

## Authors reflection

As the research team, reflecting on this co-design process, we realize the immense value of stakeholder engagement and community involvement in developing interventions that are contextually relevant and sustainable. The challenges we faced in managing diverse stakeholder interests and addressing knowledge gaps were significant, but they also presented opportunities for growth and learning. Moving forward, we would prioritize even more intentional efforts to build stakeholder capacity and foster community ownership, recognizing these as critical to the long-term impact of WASH interventions. This experience has reinforced our belief in the power of co-design approaches and shaped our perspective on navigating complexities in multi-stakeholder collaborations.

## Data Availability

The original contributions presented in the study are included in the article/supplementary material, further inquiries can be directed to the corresponding author.

## References

[1] WHO. WHO Global Water, Sanitation and Hygiene: Annual Report 2020. Geneva: World Health Organization (2022).

[2] UNICEF. Evaluation of the UNICEF-supported Federal Government of Nigeria Water, Sanitation and Hygiene Programme (2014–2017). (2022). Available online at: https://www.unicef.org/nigeria/reports/evaluation-unicef-supported-water-sanitation-and-hygiene-programme-nigeria#:~:text=This%20impact%20evaluation%20was%20commissioned,achieve%20the%20SDGs%20on%20WASH

[3] WHO. WHO Water, Sanitation and Hygiene strategy 2018–2025. Geneva: World Health Organization (2019). Available online at: https://www.who.int/publications/i/item/WHO-CED-PHE-WSH-18.03

[4] UN-Habitat. World Cities Report: The Value of Sustainable Urbanization. Nairobi: UN-Habitat (2020). p. 418.

[5] Tseole NP Mindu T Kalinda C Chimbari MJ. Barriers and facilitators to water, sanitation and hygiene (WaSH) practices in Southern Africa: a scoping review. PLoS ONE. (2022) 17:0271726. doi: 10.1371/journal.pone.0271726 35917339 PMC9345477

[6] World Health Organization United Nations Children's Fund. Progress on Household Drinking Water, Sanitation and Hygiene 2000–2024: Special Focus on Inequalities. Geneva: World Health Organization (2023). Available online at: https://washdata.org

[7] WHO/UNICEF JMP. Progress on household drinking water, sanitation and hygiene 2000–2022: Special focus on gender. New York: UNICEF data (2023). Available online at: https://data.unicef.org/resources/jmp-report-2023/ (Accessed September 4, 2025).

[8] Abdulhadi R Bailey A Van Noorloos F. Access inequalities to WASH and housing in slums in low-and middle-income countries (LMICs): a scoping review. Glob Public Health. (2024) 19:2369099. doi: 10.1080/17441692.2024.2369099 38940272

[9] Prüss-Ustün A Wolf J Bartram J Clasen T Cumming O Freeman MC . Burden of disease from inadequate water, sanitation and hygiene for selected adverse health outcomes: an updated analysis with a focus on low-and middle-income countries. Int J Hyg Environ Health. (2019) 222:765–77. doi: 10.1016/j.ijheh.2019.05.004 31088724 PMC6593152

[10] U.WHO. Progress on Sanitation and Drinking Water. 2015 Update and MDG Assessment. New York: UNICEF; Geneva: World Health Organization (2015).

[11] Fewtrell L Kaufmann RB Kay D Enanoria W Haller L Colford JM. Water, sanitation, and hygiene interventions to reduce diarrhoea in less developed countries: a systematic review and meta-analysis. Lancet Infect Dis. (2005) 5:42–52. doi: 10.1016/S1473-3099(04)01253-8 15620560

[12] Shafique S Bhattacharyya DS Nowrin I Sultana F Islam MR Dutta GK . Effective community-based interventions to prevent and control infectious diseases in urban informal settlements in low-and middle-income countries: a systematic review. Syst Rev. (2024) 13:253. doi: 10.1186/s13643-024-02651-9 39367477 PMC11451040

[13] Biswas D Ahmed M Roguski K Ghosh PK Parveen S Nizame FA . Effectiveness of a behavior change intervention with hand sanitizer use and respiratory hygiene in reducing laboratory-confirmed influenza among schoolchildren in Bangladesh: a cluster randomized controlled trial. Am J Trop Med Hyg. (2019) 101:1446. doi: 10.4269/ajtmh.19-0376 31701861 PMC6896855

[14] Dockx K Van Remoortel H De Buck E Schelstraete C Vanderheyden A Lievens T . Effect of contextualized versus non-contextualized interventions for improving hand washing, sanitation, and health in rural tanzania: study design of a cluster randomized controlled trial. Int J Environ Res Public Health. (2019) 16:2529. doi: 10.3390/ijerph16142529 31311186 PMC6678137

[15] Djenontin IN Meadow AM. The art of co-production of knowledge in environmental sciences and management: lessons from international practice. Environ Manage. (2018) 61:885–903. doi: 10.1007/s00267-018-1028-3 29623401

[16] Newton S Asbroek GT Hill Z Agyemang CT Soremekun S Etego SA . Maximizing community participation and engagement: lessons learned over 2 decades of field trials in rural Ghana. Emerg Themes Epidemiol. (2021) 18:19. doi: 10.1186/s12982-021-00110-7 34952613 PMC8709940

[17] Obienusi E Obikwelu M. Examination of gender-sensitive and socially-inclusive water, sanitation, and hygiene (WASH) facilities in the major markets of Anambra State, Nigeria. Socialscientia J Soc Sci Humanit. (2023) 8. Available online at: https://journals.aphriapub.com/index.php/SS/

[18] Proctor EK Powell BJ McMillen JC. Implementation strategies: recommendations for specifying and reporting. Implement Sci. (2013) 8:139. doi: 10.1186/1748-5908-8-139 24289295 PMC3882890

[19] Dunn M Glasgow R Handley K Kayondo JK Kupfer L Khrisna A . Fundamentals of implementation research. MEASURE Eval. (2012) MS-12-55. Available online at: https://www.measureevaluation.org/resources/publications/ms-12-55/at_download/document (Accessed July 21, 2016).

[20] Sáenz V Patino CM Ferreira JC. Implementation research and its role in public health and health policies. J Bras Pneumol. (2021) 47:e20210443. doi: 10.36416/1806-3756/e20210443 34878060 PMC9013525

[21] Peters DH Adam T Alonge O Agyepong IA Tran N. Implementation research: what it is and how to do it. BMJ. (2013) 347:f6753. doi: 10.1136/bmj.f6753 24259324

[22] Mbachu CO Agu CI Onwujekwe O. Collaborating to co-produce strategies for delivering adolescent sexual and reproductive health interventions: processes and experiences from an implementation research project in Nigeria. Health Policy Plan. (2020) 35(Supplement_2):ii84–97. doi: 10.1093/heapol/czaa130 33156942 PMC7646732

[23] Uneke CJ Ezeoha AE Uro-Chukwu HC. Promoting evidence-informed policymaking through capacity enhancement in implementation research for health researchers and policymakers in Nigeria: a cross-sectional study. J Educ Health Promot. (2018) 7:28. doi: 10.4103/jehp.jehp_103_17 29629389 PMC5852993

[24] Goodyear-Smith F Jackson C Greenhalgh T. Co-design and implementation research: challenges and solutions for ethics committees. BMC Med Ethics. (2015) 16:78. doi: 10.1186/s12910-015-0072-2 26573410 PMC4647576

[25] Huang KY Kwon SC Cheng S Kamboukos D Shelley D Brotman LM . Unpacking partnership, engagement, and collaboration research to inform implementation strategies development: theoretical frameworks and emerging methodologies. Front Public Health. (2018) 6:190. doi: 10.3389/fpubh.2018.00190 30050895 PMC6050404

[26] Ezeanolue EE Menson WN Patel D Aarons G Olutola A Obiefune M . Gaps and strategies in developing health research capacity: experience from the Nigeria implementation science alliance. Health Res Policy Syst. (2018) 16:10. doi: 10.1186/s12961-018-0289-x 29433577 PMC5809995

[27] Alhassan JA Gauvin L Judge A Fuller D Engler-Stringer R Muhajarine N. Improving health through multisectoral collaboration: enablers and barriers. Can J Public Health. (2021) 112:1059–68. doi: 10.17269/s41997-021-00534-3 34105113 PMC8651820

[28] Oliver S Liabo K Stewart R Rees R. Public involvement in research: making sense of the diversity. J Health Serv Res Policy. (2015) 20:45–51. doi: 10.1177/1355819614551848 25228453

[29] Sebola MP Mahlatji SE. Planning and implementation of government strategy for projects in the Limpopo department of economic development, environment and Tourism, South Africa. J Hum Ecol. (2014) 46:1–9. doi: 10.1080/09709274.2014.11906700

[30] UN-Habitat. Water, Sanitation and Hygiene (WASH) in Urban Informal Settlements. Nairobi: UN-Habitat (2016).

[31] Paasche T Whelan M Nahirney M Olemshumba S Bastien S. An application of the integrated behavioral model for water, sanitation and hygiene to assess perceived community acceptability and feasibility of the biosand filter among Maasai Pastoralists in rural Tanzania. Am J Trop Med Hyg. (2021) 106:464. doi: 10.4269/ajtmh.21-0398 34749313 PMC8832900

[32] Dreibelbis R Winch PJ Leontsini E Hulland KR Ram PK Unicomb L . The integrated behavioural model for water, sanitation, and hygiene: a systematic review of behavioural models and a framework for designing and evaluating behaviour change interventions in infrastructure-restricted settings. BMC Public Health. (2013) 13:1015. doi: 10.1186/1471-2458-13-1015 24160869 PMC4231350

[33] Kirk MA Kelley C Yankey N Birken SA Abadie B Damschroder L . Systematic review of the use of the consolidated framework for implementation research. Implement Sci. (2015) 11:72. doi: 10.1186/s13012-016-0437-z PMC4869309 27189233

[34] De Silva MJ Breuer E Lee L Asher L Chowdhary N Lund C . Theory of change: a theory-driven approach to enhance the medical research council's framework for complex interventions. Trials. (2014) 15:267. doi: 10.1186/1745-6215-15-267 24996765 PMC4227087

[35] Roberts R. Local Governance Strategies of Okpoko Community in Onitsha to Access Water. Abuja: Heinrich Böll Stiftung (2021).

[36] Iloerika-Okafor AC Uwadiegwu BO Onuoha DC. Assessment of the impact of slum environment on residents of Okpoko slum. Trop Built Environ J. (2024) 9. Available online at: www.tbejournal.com

[37] Samuel O Nkiruka I Frederick O. Bacteriological quality assessment of borehole water in Ogbaru communities, Anambra state, Nigeria. Univ J Clin Med. (2019) 7:1–10. doi: 10.13189/ujcm.2019.070101

[38] Braun V Clarke V. Using thematic analysis in psychology. Qual Res Psychol. (2006) 3:77–101. doi: 10.1191/1478088706qp063oa

[39] WHO. Water, Sanitation and Hygiene in Health Care Facilities. Geneva: World Health Organization (2019). Available online at: https://iris.who.int/server/api/core/bitstreams/8897e5b4-8bde-44f7-8bc0-74b61f27de7c/content

[40] Mbuya MN . Using local evidence to inform decision-making in water, sanitation, and hygiene (WASH) programs: a case study from rural Tanzania. BMC Public Health. (2021) 21:1–12.33388037

[41] WHO. Drinking-Water Quality Surveillance and Monitoring. Geneva: World Health Organization (2019).

[42] Sanka E. Impact of stakeholder engagement on the success of water, sanitation, and hygiene projects in Babati, Tanzania. J Policy Dev Stud. (2024) 17:46–53. doi: 10.4314/jpds.v17i1.4

[43] Vargas C Zorbas C Longworth GR Ugalde A Needham C Sunil A . Exploring co-design: a systematic review of concepts, processes, models, and frameworks used in public health research. J Public Health. (2025) 47:e616–e639.Available online at: https://academic.oup.com/jpubhealth/advance-article/doi/10.1093/pubmed/fdaf084/8232581 40796276 10.1093/pubmed/fdaf084 PMC12669994

[44] Ogundeko-Olugbami O Ogundeko O Lawan M Foster E. Harnessing data for impact: Transforming public health interventions through evidence-based decision-making. World J Adv Res Rev. (2025) 25:2085–103. doi: 10.30574/wjarr.2025.25.1.0297

[45] DeSouza D. Evidence-based approach to decision making: the inclusion of GIS as part of Ghana's health information systems. Ghana Med J. (2009) 43:1. 19652747 PMC2709165

[46] Chowdhury RH. Big data analytics in the field of multifaceted analyses: A study on “health care management”. World J Adv Res Rev. (2024) 22:2165–72. doi: 10.30574/wjarr.2024.22.3.1995

[47] Attah RU Gil-Ozoudeh I Garba BM Iwuanyanwu O. Leveraging geographic information systems and data analytics for enhanced public sector decision-making and urban planning. Magna Sci Adv Res Rev. (2024) 12:152–63. doi: 10.30574/msarr.2024.12.2.0191

[48] Singh UB Rade K Rao R Kumar N Mattoo SK Nair S . Lessons and updates from India's National TB Elimination Program–bold decisions and innovative ways of fast-tracking progress towards ending TB. IJID Regions. (2025). doi: 10.1016/j.ijregi.2025.100599 PMC11973646 40201556

[49] Okolo CA Chidi R Babawarun O Arowoogun JO Adeniyi AO. Data-driven approaches to bridging the gap in health communication disparities: A systematic review. World J Adv Res Rev. (2024) 21:1435–45. doi: 10.30574/wjarr.2024.21.2.0591

